# Supplementary material for: Quantitative analysis of the ThrbCRM1-centered gene regulatory network
Source: Biol Open. 2019 Apr 10;8(4):bio039115. doi: 10.1242/bio.039115 (PMC6504004; doi:10.1242/bio.039115)
Supplement: Supplementary information [file biolopen-8-039115-s1.pdf]

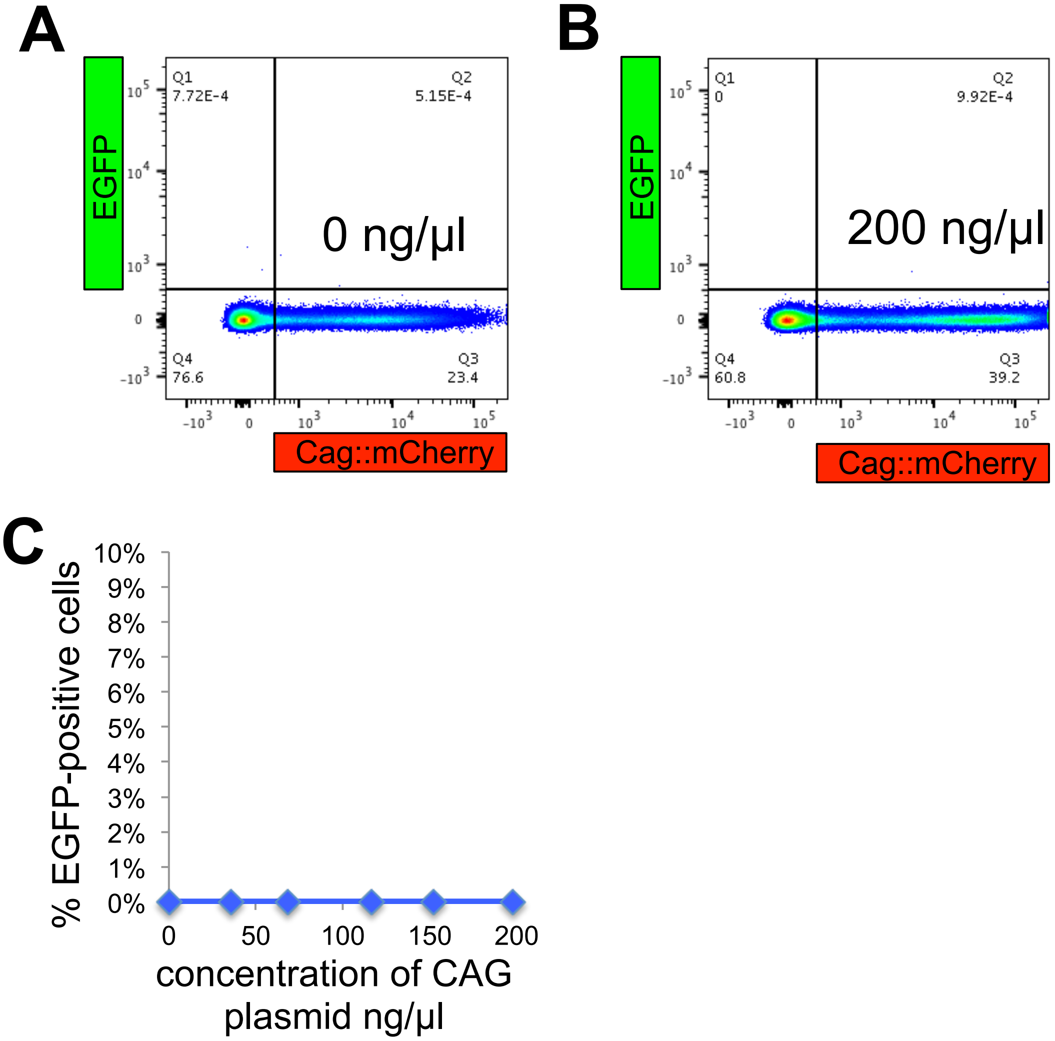

Biology Open • Supplementary information

Figure S1. Assessment of plasmid transactivation on Stagia3 reporter plasmids. (**A-B**) Representative flow cytometry plots of dissociated cells from chicken retinas electroporated with an empty Stagia3 reporter plasmid, a CAG::mCherry co-electroporation control, and another plasmid containing either 0 ng/µl (A) or 200 ng/µl (B) of an additional plasmid containing the CAG element without a fluorescent readout. Stagia3 reporter activity is plotted along the y-axis and the CAG::mCherry co-electroporation control along the x-axis. (**C**) Quantification of Stagia3 reporter plasmid activity plotted along the y-axis as the percentage of EGFP-positive in the electroporated population in the presence of varying concentrations of CAG co-electroporated plasmid in nanograms/microliter along the x-axis. Error bars represent 95% confidence intervals. There was no statistically significant effect of any concentration of additional CAG plasmid concentration compared to the baseline as assessed by a one-way Anova with a post-hoc Dunnetts test.

Biology Open • Supplementary information
